# A Brücke–Bartley effect for contrast

**DOI:** 10.1098/rsos.180171

**Published:** 2018-08-15

**Authors:** Joshua A. Solomon, Christopher W. Tyler

**Affiliations:** Centre for Applied Vision Research, City, University of London, London EC1V 0HB, UK

**Keywords:** psychophysical function, transducer function, brightness, apparent contrast, flicker

## Abstract

Accurate derivation of the psychophysical (a.k.a. transducer) function from just-notable differences requires accurate knowledge of the relationship between the mean and variance of apparent intensities. Alternatively, a psychophysical function can be derived from estimates of the average between easily discriminable intensities. Such estimates are unlikely to be biased by the aforementioned variance, but they are notoriously variable and may stem from decisional processes that are more cognitive than sensory. In this paper, to minimize cognitive pollution, we used amplitude-modulated contrast. As the spatial or temporal (carrier) frequency increased, estimates of average intensity became less variable across observers, converging on values that were closer to mean power (i.e. contrast^2^) than mean contrast. Simply put, apparent contrast increases when physical contrast flickers. This result is analogous to Brücke's finding that brightness increases when luminance flickers. It implies an expansive transduction of contrast in the same way that Brücke's finding implies an expansive transduction of luminance.

## 1. Background

No one questions the idea that visual salience increases with stimulus contrast. The question is how. Stevens [1] suggested a power-law relationship for all prothetic continua (of which, contrast is one). Thus, salience *ψ* should be proportional to *S^n^*, where *S* represents stimulus contrast and the exponent *n* is derived from psychophysical data. Stevens himself did not collect data on the power-law exponent for contrast, but many others adapted one of his scaling paradigms for this purpose. For example, Franzén & Berkley [2] reported values of *n* between 0.6 (for low-frequency gratings) and 1.7 (for high-frequency gratings). Cannon [3], on the other hand, reported values near 1.0 for all spatial frequencies. Gottesman *et al*. [4] championed 0.7, when near-threshold contrasts were ignored.

The extreme variability in these findings is hard to ignore. Laming [5] argued that it is much too great to be attributed to sensory factors and must instead be attributed to procedural details that introduce ‘contextual’ (i.e. non-sensory) biases. By way of contrast (no pun intended), there is significantly less inter-laboratory variability in estimates of the transducer function for contrast when those estimates are derived from suprathreshold contrast discrimination. With one notable exception [6], just-notable differences rise with the 0.6 or 0.7 power of contrast (e.g. [7]). Legge & Foley [8] showed that this relationship would imply a psychophysical exponent with a value near 0.4 (i.e. 1–0.6) if discrimination were limited by a source of constant noise, i.e. a noise whose variance was independent of contrast.

Unfortunately, we seem to have very little evidence supporting the idea of constant noise. One experiment specifically designed to elucidate this issue [9] concluded in favour of a performance-limiting source of noise that increased with suprathreshold contrast. Consequently, it remains premature to accept contrast discrimination data as evidence for compressive transduction (i.e. *n* < 1).

Kulikowski [10] used the fractionation paradigm to evade the complication of sensory noise: observers adjusted one grating's contrast until it seemed to be exactly half that of another, otherwise identical grating. However, this method also produced large individual differences. Possible reasons for these differences include uncertainty regarding the definition of contrast and the lack of any perceptual experience corresponding to half contrast. Cognitive influences were unavoidable.

Wu *et al*. [11] were interested in the transduction from (physical) lightness to (perceived) brightness. Like Kulikowski [10], they used the method of adjustment to investigate transducer shape. However, unlike Kulikowski, they gave their observers a specific perceptual experience to report by means of a bipartite stimulus. One half of the stimulus was flickering. Its temporal (carrier) frequency was one of the independent variables. Nonlinear transduction creates a distortion product in flickering stimuli. Whereas a compressive transducer would reduce the mean level of modulated input, high amplitude flicker looks, on average, disproportionately bright. This Brücke–Bartley effect implies an expansive transducer (i.e. *n* > 1), which disproportionately amplifies large input. Wu *et al*. [11] sinusoidally modulated flicker amplitude at 0.5 Hz. The other half of their stimulus was an otherwise steady light, whose luminance was modulated at 0.5 Hz. Observers adjusted the latter modulation until it appeared to match the distortion product.

For Experiment 1, we have adapted the procedure of Wu *et al*. (a similar method was used by Petrova *et al*. [12]) for an investigation of contrast transduction. Before any data were collected, we created several stimuli (three of which have been included with the electronic supplementary material) demonstrating both the Brücke–Bartley effect and its contrast analogue. In Experiment 2, we investigated the effect of spatial modulations on average salience, to see if they too were consistent with the same power-law transducer.

## 2. Methods

### 2.1. Stimuli

Our demonstration stimuli should work on any gamma-corrected monitor, but our data were collected using a Sony GDM-F520 connected to a MacBook Pro via Cambridge Research Systems' Bits#. Resolution was 640 × 480 × 120 Hz. Background luminance was held constant, midway between the maximum (i.e. greylevel 1: 153.8 cd m^−2^) and minimum (i.e. greylevel 0: 42.57 cd m^−2^) luminances. Consequently, no pixel could attain a Weber contrast *C* outside the range (−0.57, 0.57).

The room was darkened, so that most of its light came from the stimulus monitor, although a minor contribution came from this light reflected off the laboratory's other contents. The monitor's viewable size was 50.3 cm. Viewing was binocular. We did not use artificial pupils nor did we enforce fixation.

All of our stimuli are defined as the product of a rather complicated angular modulator (equation (2.1)) and the Weber contrasts of a very simple spatial annulus. Prior to angular modulation, one of these annuli was uniformly bright (i.e. *C*_0_ = 0.57), another was uniformly dark (i.e. *C*_0_ = −0.57), and in the third each 2 × 2 block of pixels was randomly assigned to one of these two values. Using 2 × 2 blocks of pixels (rather than individual pixels) helps to reduce any distortion products due to the Sony's limited bandwidth.^1^ At the observers' 1.6 m viewing distance (Experiment 1), the inner and outer radii of each annulus subtended 0.5° and 1.0° of the visual angle, respectively. Larger viewing distances were used in Experiment 2: 3.6 and 10.9 m. The angular subtenses reduced proportionately.

Under separate conditions, one of the three basic annuli was scaled, such that its Weber contrasts became a function of time *t* and angle *θ* (the latter of which was calculated anti-clockwise from the bottom or the top, in alternating blocks of trials):

2.1 $$C(t,\theta )=\left\{\begin{array}{ll} \frac{wC_{0}}{8}\left[4+\left(\cos\left[2\pi \left(\frac{t}{t_{c}}+\frac{\theta }{\theta_{c}}\right)\right]+\cos\left[2\pi \left(\frac{t}{t_{c}}-\frac{\theta }{\theta_{c}}\right)\right]\right)(1+\cos2\theta )\right] & \cos\theta >1\\ \frac{wC_{0}}{2}\left[1+\frac{a}{2}(1+\cos2\theta )\right] & \cos\theta \leq 1 \end{array}\right..$$

We will use the terms ‘standard’ and ‘adjustable’ to describe the two halves of each annulus, which meet at the horizontal meridian. The standard half underwent counterphasing modulation with temporal and angular wavelengths *t*_c_ and *θ*_c_, respectively. To avoid a sharp discontinuity at the transition between the two regions, the envelope of the carrier's amplitude was defined by an angular sinewave, with period *π*, such that its peak occurs where *θ* = 0 (i.e. at the top or bottom). Contrasts in the adjustable half-annulus were also described by an angular sinewave with period *π*, but the amplitude of this sinewave (parameter *a*) was under the observer's control. We refer to this parameter as the adjuster. Finally, parameter *w* established the average amplification of Weber contrasts. In order to avoid Weber contrasts outside the range of our apparatus, parameters *a* and *w* were constrained, such that −1 ≤ *a* ≤ *w*^−1^. In all the experiments reported below, we fixed *w* = 0.5.

**Figure 1.**
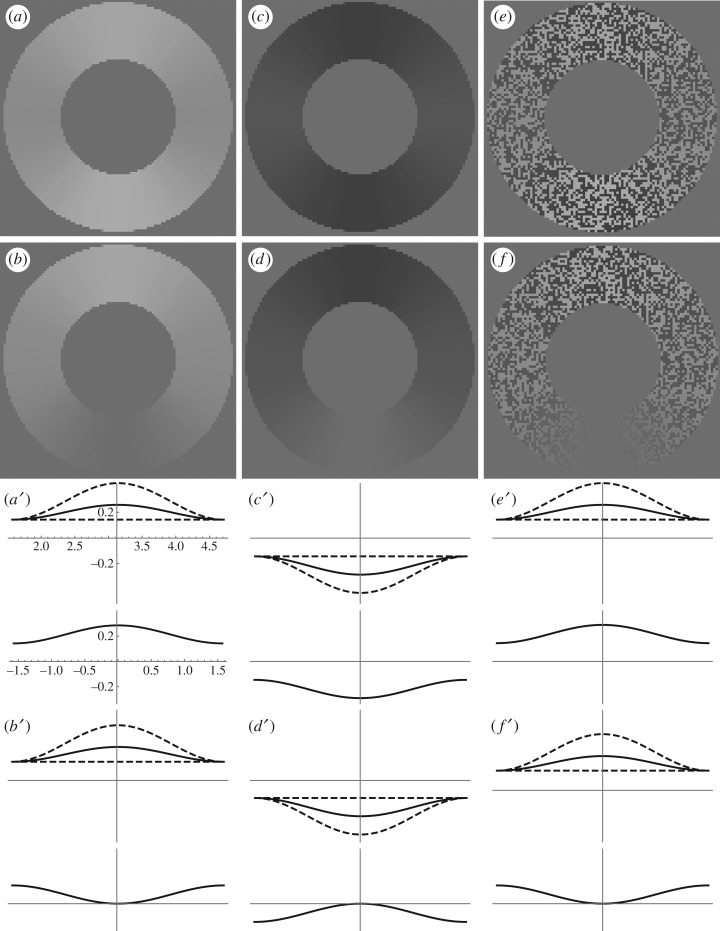
Example stimuli from Experiment 1. Flickering between panels (*a*) and (*b*) produces the Brücke–Bartley effect (the bottom of this bright annulus appears brighter than its sides, even though they have the same average luminance). When the flicker was sinusoidal, with a frequency of 7.5 Hz, our observers judged the (static) top of this bright annulus to be a good match for the bottom's average brightness. Flickering between panels (*c*) and (*d*) makes the bottom of this dark annulus appear darker than its sides. Note: in both cases, flicker increases the average salience. In this paper, we report the analogous result with random texture. Flickering between panels (*e*) and (*f*) makes the bottom of this textured annulus more salient than its sides. Each of the panels (*a*′–*f*′) contains two graphs of the function (solid curve) mapping angle to Weber contrast (or, in the cases of panels (*e*′) and (*f*′), absolute Weber contrast) in the corresponding panels (*a*–*f*). Panel (*a*′) illustrates the domains (bottom graph: [−*π*/2, *π*/2]; top graph: [*π*/2, 3*π*/2]) and range (all graphs: [–0.43, 0.43]) common to all panels (*a*′–*f*′). Dashed curves show otherwise identical functions with the parameter *a* fixed at the values 0 and 2.

Each online demo (a, b and c) contains one of the three basic annuli (bright, dark and textured, respectively) with *a* = 0, $\theta_{c}^{-1}\approx 0$ (more precisely, we set *θ*_c_ = 10^6^), and $t_{c}^{-1}=7.5\;\text{Hz}$. The Brücke–Bartley effect and its contrast analogue should be apparent with this frequency. For figure 1, we have adjusted *a* to match the averages of our observers’ adjustments in Experiment 1 (i.e. with a carrier frequency of 7.5 Hz in Conditions 1–3). Figure 1 shows two frames from each stimulus: one with the largest product of carrier and modulator (e.g. *t* = 0) and one with the smallest product (e.g. *t* = *t*_c_/2).

### 2.2. Observers and procedure

Both authors confirmed the appearance of a distortion product, as described in the legend of figure 1. Author J.A.S. and four other experienced psychophysical observers (M.L., J.F., M.C. and P.C.) generated data. At the beginning of each trial, the adjuster (*a* in equation (2.1)) was given a random value between −1 and 1. In Conditions 1–3 (corresponding to the three basic annuli: bright, dark and textured) observers were instructed to adjust the adjuster until average salience (also called ‘brightness’ in Condition 1, ‘darkness’ in Condition 2, and ‘contrastiness’ in Condition 3) at the bottom of the annulus was equal to that at the top.^2^ The adjuster decreased by 0.05 with each press of the ‘c’ key and increased by the same amount with each press of ‘m’. At no time were observers informed of the adjuster's value. When satisfied with their adjustment, observers initiated a new trial by depressing the space bar.

Conditions 4–6 used the same stimuli, but different instructions. In these conditions, observers were instructed to adjust the adjuster until the maximum salience at the bottom of the annulus was equal to that at the top. The rationale behind Conditions 4–6 will be discussed below. Within each condition, five carrier frequencies were tested (see below). For each combination of condition and carrier frequency, each observer completed two successive blocks of three trials each; one with *θ* = 0 at the top of the annulus (as in figure 1), and one with *θ* = 0 at the bottom.

In Experiment 1, we tested temporal modulations; the carrier had an angular frequency $\left(\theta_{c}^{-1}\right)$ of zero and a temporal frequency $\left(t_{c}^{-1}\right)$ of 3.75, 7.5, 15, 30 or 60 Hz. This latter frequency is one half of the refresh rate of our monitor, and exceeds the critical fusion frequency of the visual system. In other words, 60 Hz flicker (around a mean luminance of 98.2 cd m^−2^) is imperceptible. The white, black and textured annuli appeared to be completely static.

**Figure 2.**
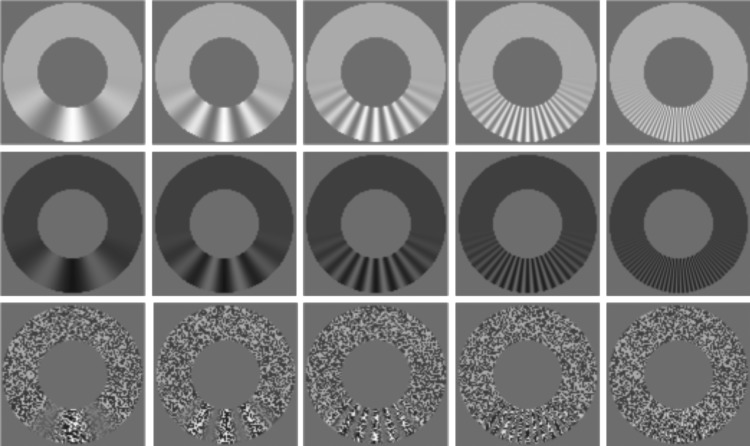
Stimuli from Experiment 2. In each panel, *w* = 1 and *a* = 0.

In Experiment 2, we tested spatial modulations in the same general format as for Experiment 1; the carrier had a temporal frequency $\left(t_{c}^{-1}\right)$ of zero and angular frequencies of *π*/3, *π*/6, *π*/12, *π*/24 or *π*/48, as illustrated in figure 2. The latter frequency was achieved using exactly 2 pixels per cycle on the inner edge of the annuli. As noted above, viewing distances were increased to ensure that the highest of these frequencies was beyond the spatial resolution of the visual system. Mirrors were employed so that light from the black and white annuli travelled 10.9 m to the observer's eyes. However, at this great distance, the textured annuli were too hard to see. Thus, for the textured annuli, we used a viewing distance of 3.6 m, which proved sufficient to prevent detection of the highest frequency of contrast modulations.

## 3. Experiment 1: results

**Figure 3.**
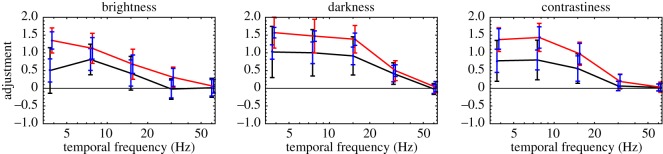
Summary of Experiment 1 results. Black curves connect average adjustments matching average salience (i.e. Conditions 1–3). Red curves connect average adjustments matching maximum salience (i.e. Conditions 4–6). Colour-coded error bars contain two SDs, when all observers' data were pooled (i.e. across observers). Blue error bars contain two average SDs (i.e. within observers, over trials).

Applying Bonferroni correction for multiple comparisons, we used Grubbs' statistic to test the (null) hypothesis of no outliers at the *α* = 0.05 level of significance. On this basis, none of the (6 trials per condition per frequency per observer × 6 conditions × 5 frequencies × 5 observers) 900 trials were deemed to be outliers. Results are summarized in figure 3. (Corresponding figures for individual observers have been made available in the electronic supplementary material.)

Whether bright, dark or textured, at 60 Hz (i.e. $t_{c}^{-1}=60\;\text{Hz}$), all flickering annuli appeared to be steady. In all six conditions, the average adjustment at this frequency was not significantly different from zero, according to one-sample, two-tailed *t*-tests (*p* > 0.47 in all cases). After Bonferroni correction for multiple comparisons, one-way analyses of variance (ANOVA) at the *α* = 0.05 level of significance indicated no individual differences in any of the six conditions at this frequency.

### 3.1. Condition 1 (average brightness)

When queried, all observers reported that flicker was visible at 30 Hz. However, our data contain no evidence for a Brücke–Bartley effect at this frequency (i.e. adjuster settings were insignificantly greater than zero, both when individual observers’ data and all data combined were subjected to one-sample, one-tailed *t*-tests; in all cases, *p* > 0.05). This result is consistent with previous reports of the relatively narrow tuning of the Brücke–Bartley effect [11,12].

The Brücke–Bartley effect was visible to both authors at 15 Hz. Consequently, observers were expected to set the adjuster at some positive value, effectively increasing luminance in the non-flickering, adjustable half of the annulus. Indeed, when all data are combined, the mean was significantly greater than zero, according to a one-sample, one-tailed *t*-test (*p* < 10^−7^). However, some observers' adjustments were too variable for their data to pass the same *t*-test on an individual basis.

Individual differences (i.e. between observers) were even more pronounced with 7.5-Hz flicker (*p* < 0.0005, according to a one-way ANOVA). However, when all data were combined, their mean was even greater than that at 15 Hz. Data from 3.75 Hz mirrored those from 15 Hz: when all are combined, the mean is significantly greater than zero (*p* < 10^−4^); however, there were significant differences between individuals’ average settings (one-way ANOVA, *p* < 10^−5^).

This pattern of results suggests that the Brücke–Bartley effect is maximal at frequencies near 7.5 Hz under our observation conditions. Similarly, Bartley [13] reported the largest effects with frequencies between 8 and 9 Hz. However, Brücke's [14] original effect was obtained using 17.5 Hz modulation, and Wu *et al*. [11] obtained their maximum effect at 16 Hz. Consequently, it seems safe to conclude that the Brücke–Bartley effect is quite robust, but the flicker rate optimal for eliciting it seems subject to considerable individual differences and/or variations in the experimental conditions. Our stimuli for the brightness and darkness conditions, for example, were essentially foveal (within 1° eccentricity with central fixation, or 2.5° with fixation in the annulus), whereas Wu *et al*. employed a 10° field which would be expected to have substantially higher temporal resolution [15,16].

### 3.2. Condition 2 (average darkness)

To a large extent, our observers' adjustments with respect to average darkness parallel their adjustments with respect to average brightness. At each frequency below 60 Hz, the average adjustment was significantly greater than zero (*p* < 10^−7^). (Online Demo **b** shows a 7.5 Hz example with the adjuster fixed at 0.) One-way ANOVAs indicated significant (*p* < 0.01) individual differences for all carrier frequencies except 60 Hz.^3^

### 3.3. Condition 3 (average contrastiness)

To a large extent, our observers’ adjustments with respect to average contrastiness parallel their adjustments with respect to average brightness and average darkness. At 30 Hz and below, the standard half of the annulus (where *θ* = 0) appeared to have greater contrast. This exaggerated salience, which we dub the ‘Brücke–Bartley effect for contrast’, was reflected in the data. Average adjustments were largest with 7.5 Hz flicker, but significantly (*p* < 0.01) greater than zero for all frequencies except 60 Hz.

As with average brightness and average darkness, the variance of adjustments (both within and between observers) was particularly large when the carrier frequency was 3.75 Hz (note the large error bars in figure 3). We hypothesized that our observers had no clear perceptual experience corresponding to the average salience of a slowly modulating stimulus. Conditions 4–6 were designed because some observers felt more comfortable deciding whether the top or bottom of any slowly flickering annulus attained greater peak or maximum salience than when deciding whether the top or bottom had a greater mean. The results for these conditions, described in the next three sections, are shown by the red curves in figure 3.

### 3.4. Condition 4 (maximum brightness)

At all frequencies below 60 Hz, average adjustments in Condition 4 were higher than those in Condition 1. This was to be expected because flicker is visible at these frequencies, and the peak luminance of a flickering light must logically be brighter than its average luminance. Further comparisons between Conditions 1 and 4 revealed no other systematic effects. Although Condition 4 felt more natural than Condition 1 to at least three of the five observers, this feeling was not reflected in systematically lower SDs of adjustment.

### 3.5. Condition 5 (maximum darkness)

Observers’ adjustments with respect to maximum darkness largely paralleled their adjustments with respect to maximum brightness. For the three lowest carrier frequencies, mean adjustments were higher than the corresponding adjustments in Condition 2 (*p* < 10^−5^ in all cases); and thus also significantly greater than zero. Furthermore, as in Condition 2, observers in Condition 5 were different in their average settings. One-way ANOVAs indicated significant (*p* < 10^−5^) individual differences at all frequencies except 60 Hz.

### 3.6. Condition 6 (maximum contrastiness)

For all carrier frequencies below 60 Hz, average adjustments were greater than the corresponding settings in Condition 5 (*p* < 0.01 in all cases), and thus also greater than zero.

## 4. Experiment 2: results

**Figure 4.**
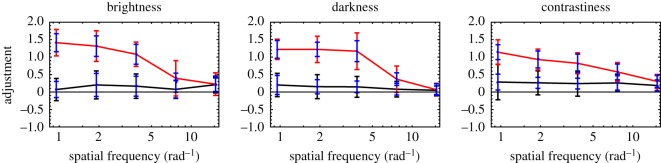
Summary of Experiment 2 results. Black curves connect average adjustments matching average salience (i.e. Conditions 1–3). Red curves connect average adjustments matching maximum salience (i.e. Conditions 4–6). Colour-coded error bars contain two SDs, when all observers’ data were pooled (i.e. across observers). Blue error bars contain two average SDs (i.e. within observers, over trials).

Experiment 2 was similar to Experiment 1 except that the (static) carrier gratings were defined by their angular frequencies rather than by their temporal frequencies. Once again, applying Bonferroni correction for multiple comparisons, we used Grubbs' statistic to test the (null) hypothesis of no outliers at the *α* = 0.05 level of significance. On this basis, none of the 900 trials could be deemed an outlier. Results are summarized in figure 4. (Corresponding figures for individual observers have been made available in the electronic supplementary material.)

Although no stripes were visible at the highest angular frequency (i.e. $\theta_{c}^{-1}=48/\pi$), when all observers’ data were pooled, the average adjustment in Condition 1 (Average Brightness) was 0.21 ± 0.04, significantly different from zero according to a one-sample, two-tailed *t*-test (*p* < 10^−4^). Indeed, after Bonferroni correction for multiple comparisons, only one of the black curves (the Darkness curve from Condition 2, at 48/π) deviated significantly (i.e. *p* < 0.05/15) from their shared average value (of 0.17).

At the highest angular frequency (i.e. $\theta_{c}^{-1}=48/\pi$), mean adjustments (in Conditions 1 and 2) and maximum adjustments (in Conditions 4 and 5) were insignificantly different (in all cases, *p* > 0.3), according to a two-way ANOVA over factors Condition and Observer. At all other frequencies, mean and maximum adjustments were significantly different (*p* < 0.01 in all cases).

## 5. Modelling

Before adapting the methodology of Wu *et al*. [11] for an investigation of contrast transduction (i.e. in Condition 3), we first (in Experiment 1, Condition 1) ensured that these methods were suitable for measuring the traditional Brücke–Bartley effect. Accordingly, it seems sensible to review how the model of Wu *et al*. [11] (also used by Petrova *et al*. [12]) applies to the traditional effect, before attempting to apply it to our contrast data.

Adopting the original terminology of Spekreijse & Reits [17], Wu *et al*. [11] called their model a ‘sandwich’. In such a model, incoming luminance is initially convolved with a temporal kernel that is responsible for the band limit on salience of the distortion product. The distortion product itself is caused by the middle of the sandwich: an instantaneous (expansive) nonlinear transducer. Finally, the filtered, transduced signal is convolved with yet another kernel, whose relatively long time constant serves to erase high frequencies from salience. Like Wu *et al*. [11], we did not systematically measure flicker fusion frequencies (cf. [12]), thus we can only make broad generalizations about the high-frequency cut-off of this second filter. Nevertheless, we know that it does not pass 60 Hz, because flicker at that frequency was completely invisible.

To complete the psychophysical model, we need to establish adjustment criteria. Adjustments towards a perceptual average (as in Conditions 1–3) seem likely to minimize the root-mean-square (RMS) difference between the top and bottom of the annulus, after sandwich filtering. Our stimulus was designed so that, given sandwich filtering, any adjustment that minimizes the RMS difference between the top and bottom half-annuli also minimizes their mean (unsigned) difference. Adjustments towards a perceptual maximum (as in Conditions 4–6) seem likely to equate the maximum filtered signals, originating from the top and bottom of the annulus.

In order to observe the behaviour of this model, all that remains is to specify the shapes of the filters and nonlinearity. For the present, we will adhere to Stevens' [1] suggestion of a simple power law. (Had we collected data using more than one value of *w*, a more complicated transducer function might have been indicated.) For the power law, any exponent *n* > 1 will produce a positive distortion product (i.e. one with relatively high salience). Similarly, we will adopt the general forms for band-pass (early) and low-pass (late) filter used by Petrova *et al*. [12].

In the (complex) frequency plane, the low-pass filter can be described by the equation

5.1 $$L(\;f;f_{L})={\left(\frac{if}{f_{L}}+1\right)}^{-2},$$

where the parameter *f*_L_ may be interpreted as the filter's corner frequency, above which the gain rapidly diminishes. Similarly, the band-pass filter can be described by the equation

**Figure 5.**
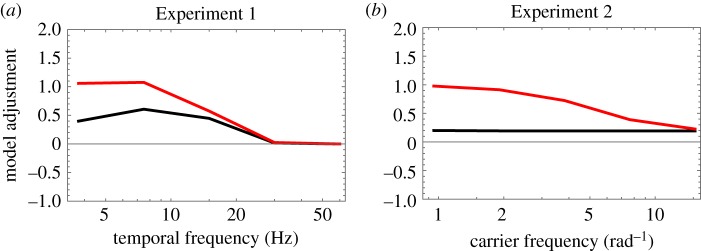
Model behaviour. Black curves connect model adjustments matching average salience. Red curves connect model adjustments matching maximum salience. (*a*) Predictions of the sandwich model for Experiment 1. Parameter values were *n* = 2, *f*_L_ = 6 Hz, *f*_C_ = 20 Hz and *f*_S_ = 5 Hz. (*b*) Predictions of a simplified model for Experiment 2. The spatial low-pass filter had parameter *f_x_* = 40 cycles per degree of visual angle.

5.2 $$B(\;f;f_{C},f_{S})={\left(\frac{iff_{C}}{+}1\right)}^{-8}/L(f;f_{S}),$$

where the parameters *f*_C_ and *f*_S_ may be interpreted as the corner frequencies of this filter's excitatory centre and (divisively) inhibitory surround components. Model behaviour is illustrated in figure 5*a*. For this proof-of-concept, no attempt was made to optimize the fit of the model to the data, but the approximating parameter values were *n* = 2, *f*_L_ = 6 Hz, *f*_C_ = 20 Hz and *f*_S_ = 5 Hz.

The parameter *n* controls the size of the distortion product. When *n* increases, the adjuster must increase accordingly. Thus, both curves (black for average salience; red for maximum salience) rise with *n*. As *n* decreases towards 1, both curves fall towards zero. (Illustrations of model behaviour with different parameter values can be found in the electronic supplementary material.) As *f*_L_ increases, so does the ratio of red-to-black heights (i.e. above zero) on the right side of the figure. That is because high-frequency flicker becomes more salient and thus its maximum and mean visibilities will differ more. Curves on the left side of the figure (i.e. for low carrier frequencies) are affected less by *f*_L_. As *f*_C_ increases, so does the high-frequency cut-off of the black curve (i.e. the frequency at which the model's adjuster drops to zero). Similarly, a low-frequency cut-off can appear with high values of *f*_S_, but the height of the black curve depends on how much DC (i.e. signal content at 0 Hz) is passed by the band-pass filter—without any DC, the unmodulated half-annulus would be invisible—and that depends on the ratio *f*_C_/*f*_S_.

Note that this sandwich model of Wu *et al*. [11] was specifically designed to describe how the visual system processes temporal modulations of stimulus luminance. In our experiments, all stimuli appeared against a uniform grey background of 98.2 cd m^−2^. Thus, any modulation of stimulus luminance was also a modulation of stimulus contrast. Consequently, when applying this model to the results of our experiments, even those from Experiment 1/Condition 1, it may be more appropriate to specify the input as modulations of stimulus contrast, rather than stimulus luminance *per se*. Moreover, the results from Experiment 1/Condition 2, in which average salience increased with luminance *decrements* from the grey background, suggest that an unsigned measure of contrast is the appropriate form of model input. Indeed, the average adjustments in these two conditions were qualitatively similar (e.g. the highest settings for average salience, made with relatively low carrier frequencies, were somewhere between 0.5 and 1; settings for average salience with high carrier frequencies were close to zero). The model behaviour illustrated in figure 5*a* shares these qualities with our results.

Average adjustments in Experiment 1/Condition 3 were also similar to the model behaviour illustrated in figure 5*a*. This should not be surprising. In Condition 1, we saw that flicker makes bright things seem brighter than unmodulated stimuli of average luminance. In Condition 2, we saw that flicker makes dark things seem darker than unmodulated stimuli of average luminance. As the textured annuli used in Condition 3 contained both bright spots and dark spots, its apparent contrast should naturally seem greater than that of an unmodulated texture of average contrast.

Now consider the results of Experiment 2. None of the average settings (across observer) differed significantly from the mean of 0.17. This result actually fits nicely with the notion that salience (contrastiness) varies with MS contrast. Figure 5*b* shows the behaviour of a simplified sandwich model (i.e. just a low-pass filter following a square-law transduction of contrast). In this case, however, the low-pass filter is specified in terms of its spatial cut-off frequency, rather than its temporal cut-off frequency. One consequence of using angular gratings is that their spatial frequencies (in cycles per degree of visual angle) vary with distance from the centre of the annulus. The model used to create figure 5*b* used the output of a radial Gaussian filter, positioned halfway between the inner and outer radii of the annulus. In frequency space, the filter fell to half-height at 47 cycles per degree of visual angle (i.e. half-height = 1.17 *f_x_*). This filter affects only the discrepancy between the red and black curves in figure 5*b*. The height of the black curve is determined wholly by the exponent *n*, in the power-function transducer. When *n* = 2 (i.e. linear transduction of contrast power), model adjustments fall between 0.19 and 0.20 for all carrier frequencies. This is quite similar to (i.e. not significantly different from) the settings our observers actually produced.

## 6. Discussion

We asked our observers to adjust an unmodulated stimulus so that it matched the average salience of a (spatially or temporally) modulated one. This is not a natural task. Individual differences arose as soon as the modulation frequencies were low enough to be detected. Nonetheless, just as Brücke [14], Bartley [e.g. 13] and many others have reported, all observers tended to elevate the difference between the adjustable stimulus and its background to exceed the average difference between the standard stimulus and its background. Our Experiment 1 demonstrates that this Brücke–Bartley effect holds not only for positive (i.e. bright) luminance differences, but also for negative (i.e. dark) luminance differences, and for the contrast differences that are the main focus of the study.

Furthermore, our modelling shows that all of our results can be fully accommodated by a sandwich model with an expansive nonlinearity; specifically, with a psychophysical function by which apparent contrast increases with the square of physical contrast (i.e. linearly with contrast power).

Although we cannot rule out the possibility that transduction depends on variables (such as the background luminance) that we did not manipulate, it really should not be surprising to find a concordance between average salience and average contrast power, especially with easily visible, spatial modulations in contrast. Although the contrasts in our textured annuli modulated around a mean value, high modulation amplitudes naturally correspond to greater RMS contrast (i.e. when the mean is calculated with respect to all phases of modulation). In other words, our observers in Experiment 2/Condition 3 behaved as though they were matching RMS contrast in the two halves of the annuli. Indeed, this behaviour could not be considered unreasonable, given their instructions to use ‘average contrastiness’ as the basis of their responses.

It may be surprising that observers seemed capable of computing RMS (or simply MS) contrast even in situations where the contrast modulations were invisible (i.e. when $\theta_{c}^{-1}=48/\pi$).^4^ If these high spatial frequencies were incapable of passing an early visual filter, then contrast averaging (i.e. Weber-contrast averaging) should have equated RMS (or MS) contrasts in both halves of the annulus, and consequently there would seem to be no basis on which to set the adjuster to any value other than 0. Accordingly, there seems to be no alternative to the implication that the front-end filter has a spatial cut-off frequency that is higher than that of the ‘down-stream’ filter that is determining the final percept, which does not pass the carrier frequency.

Previous evidence for the expansive transduction of contrast can be found in studies of contrast-defined motion perception. For example, Ukkonen & Derrington [18] noted that the motion of appropriately masked contrast modulations (but not luminance modulations) disappears around 4 Hz if the overall contrast is low. They argued that motion-processing neurons could be sensitive only to net luminance modulation, and that the distortion products of low-contrast, contrast-based translations were too weak to stimulate them; thus, the higher-order process of feature-tracking was required to reveal the contrast-defined motion.

Moulden *et al*. [19] found that different adaptors having the same RMS contrasts similarly affected a target's salience. Although this result strongly suggests that RMS contrast is an appropriate unit for the abscissa of the psychophysical function (i.e. Stevens' *S*), it does not constrain the shape of the psychophysical function in any way. Similar results would be expected if effective contrast (or contrastiness or whatever we are calling transduced contrast) were MS (not RMS) contrast. In other words, equal RMS contrast implies equal MS contrast (and vice versa).

We must accept that contrast processing may involve further nonlinearities (compressive or normalizing) which transform the visual signal after averaging has taken place. As both halves of our annuli would undergo any such transformation, its effects would not be seen in our data. Further nonlinearities of this nature were proposed by Graham & Sutter [20], who made a compelling argument in favour of a filter-rectify-filter model for texture segmentation in which the rectifying nonlinearity was expansive (a power function with an exponent ‘somewhat higher than 2’) and output of the second filter was subject to divisive normalization. Graham & Sutter's [20] putative second filter effectively computed the sum or average of the transduced energies of different micropatterns (either squares of uniform luminance or grating patches). A similar filter could play a role in some of our experiments, where observers were asked to make decisions on the basis of the average salience of a stimulus with spatially or temporally modulated energy.

Both our results and our conclusions resemble those of Meese *et al*. [21], whose observers were required to match the ‘overall’ (or ‘global’) contrast of heterogeneous texture elements with that of a homogeneous texture. Matches were made when the contrast of the uniform texture was perceived as somewhere between the average and maximum contrast of the heterogeneous texture, a result consistent with expansive transduction of contrast (with an exponent of 2.4) prior to the addition of heterogeneous signals.

Despite some individual differences, all observers experienced the analogue of the Brücke–Bartley effect for contrast. At no time did any of our observers ever produce adjustments that were significantly less than zero,^5^ which would have suggested compressive transduction. Our observers' positive adjustments lead us to conclude that contrast undergoes expansive transduction, producing distortion products that increase average salience when image brightness, darkness, or contrast is modulated in time or space.

## Supplementary Material

Individual data

## Supplementary Material

Model Behaviour

## Supplementary Material

Movie demo A

## Supplementary Material

Movie demo B

## Supplementary Material

Movie demo C

## Footnotes

1 On this monitor, vertical, squarewave, luminance gratings were darker than otherwise identically constructed horizontal gratings when their wavelength was 2 pixels. No such difference in luminance was detectable when wavelengths were increased to 4 pixels.

2 Explicit, written instructions were, ‘Use the keys c and m to reduce and increase visibility (brightness, darkness or contrastiness) at the top or bottom of the annulus. Press the space bar when top and bottom have either equal average visibilities (Task 2) or equal maximum visibilities (Task 3) when fixating the centre.’ Task 2 is our Experiment 1. Each observer completed Task 2 before beginning Task 3. Task 3 is our Experiment 2. There was no Task 1.

3 Experiment 1, Conditions 2 and 5 produced the largest ratios of SD (pooled across observers: average within observers), i.e. the most egregious failures of consensus among our observers. We are not sure why.

4 The reader is encouraged to place figure 2 at the distance (or just beyond it) where the carrier modulation in the lower right panel becomes invisible, and verify whether the bottom of the annulus does appear to have slightly greater salience than the top.

5 Observer PC came closest with a 60 Hz carrier in Experiment 1/Condition 1. A one-tailed *t*-test suggested the *p*-value of 0.018, but that would not pass Bonferroni correction for multiple comparisons.

## Data accessibility

Analyses (1 Mathematica file), Raw data (41 text files) and code (1 Matlab file) are available at http://www.staff.city.ac.uk/~solomon/dataandcodeforPRS.zip. The code uses PsychToolbox [22,23] sub-routines.
