# Supplementary material for: A Brücke–Bartley effect for contrast
Source: R Soc Open Sci. 2018 Aug 15;5(8):180171. doi: 10.1098/rsos.180171 (PMC6124126; doi:10.1098/rsos.180171)
Supplement: Individual data [file rsos180171supp1.pdf]

Each row of panels below has the same format as Figs. 3 and 4. Black curves connect average adjustments matching average visibility (i.e. Conditions 1-3). Red curves connect average adjustments matching maximum visibility (i.e. Conditions 4-6). Vertical axes are doubler values. Error bars contain 2 SDs (i.e., over trials).

Experiment 1: Temporal

JS

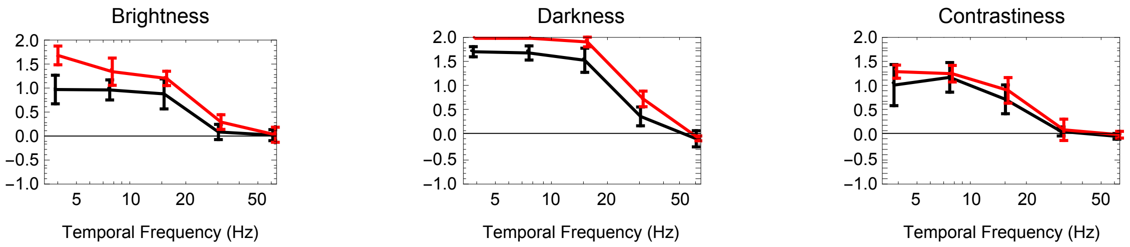

ML

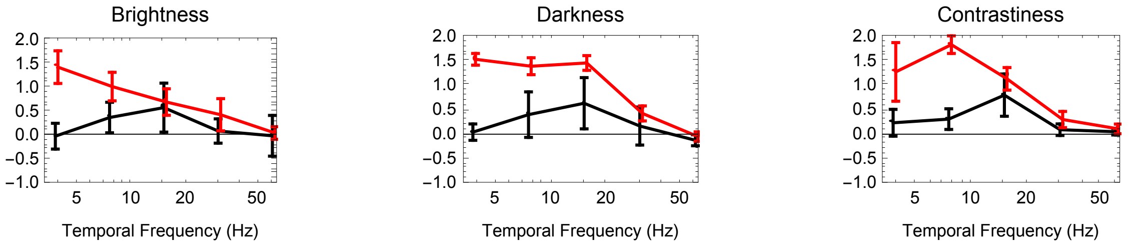

JF

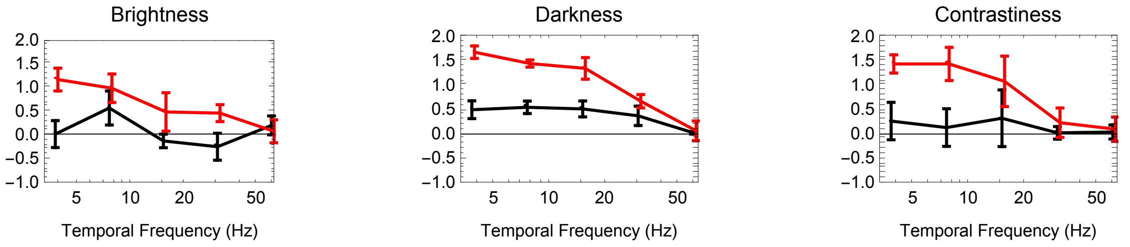

MC

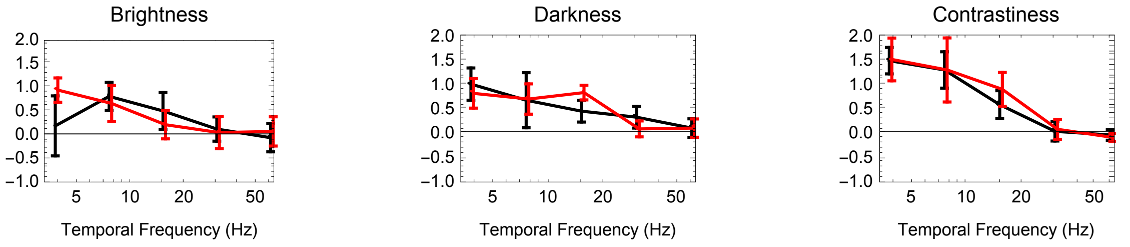

PC

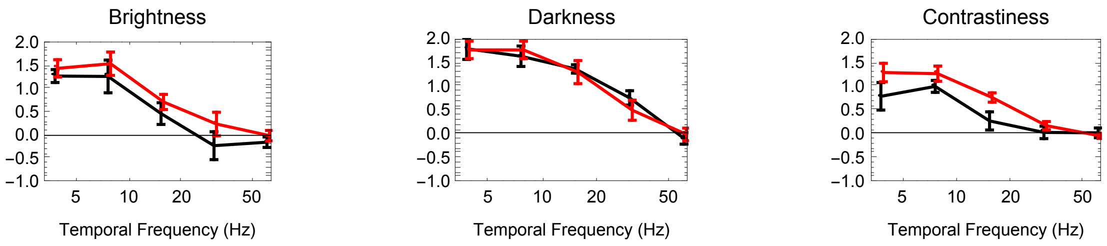

Experiment 2: Spatial

JS

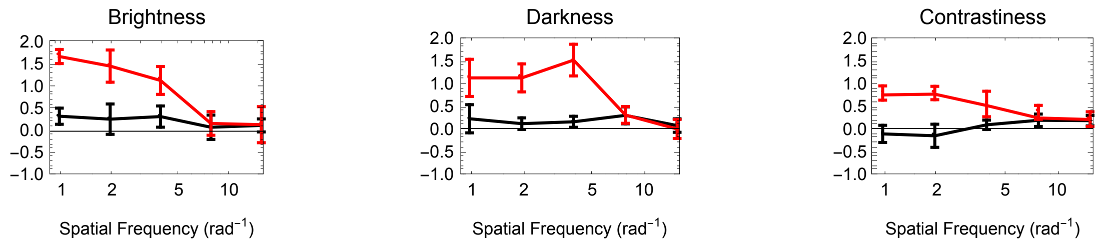

ML

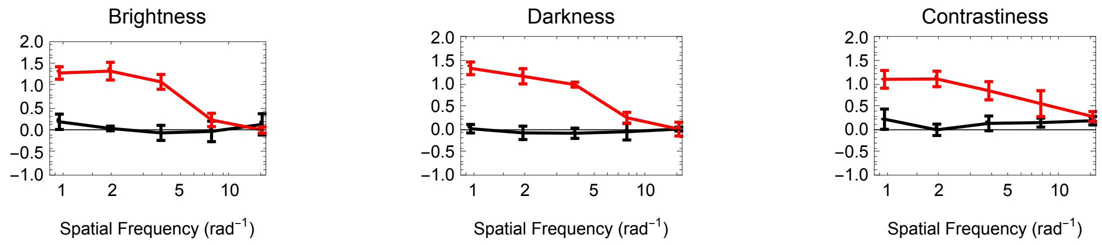

JF

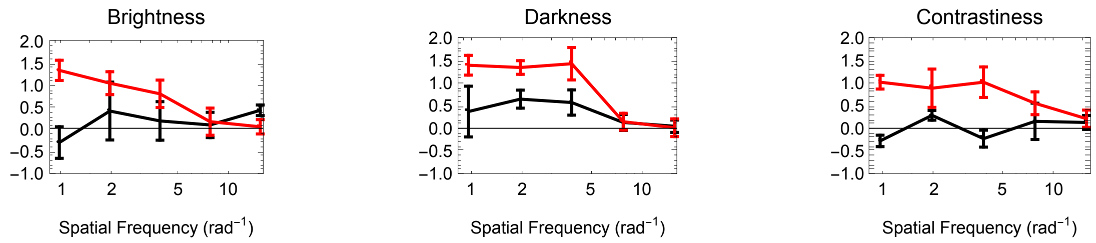

PC

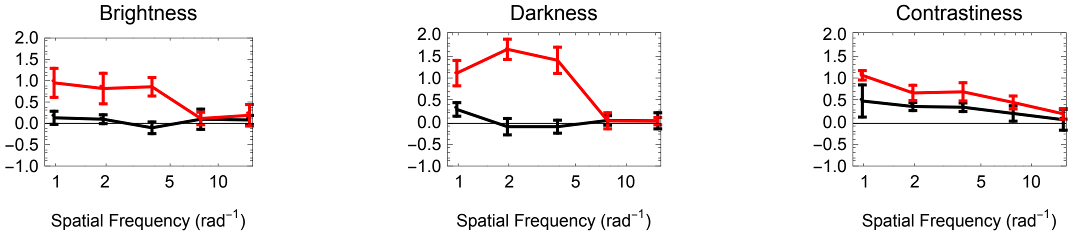
Brightness
Darkness
Contrastiness
2.0
1.5
1.0
0.5
0.0
−0.5
−1.0
1
2
5
10
Spatial Frequency (rad−1)

MC

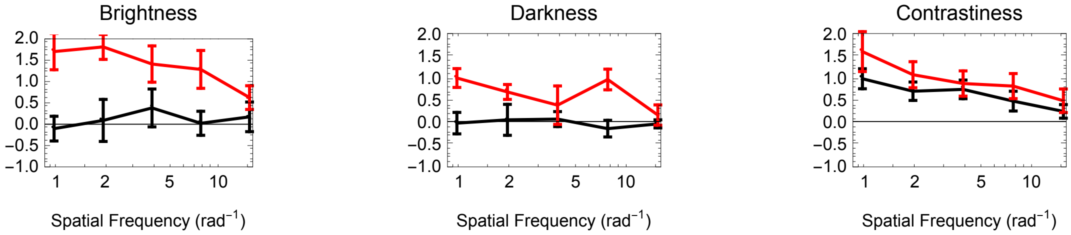
Brightness
Darkness
Contrastiness
2.0
1.5
1.0
0.5
0.0
−0.5
−1.0
1
2
5
10
Spatial Frequency (rad−1)
