# Supplementary material for: A Brücke–Bartley effect for contrast
Source: R Soc Open Sci. 2018 Aug 15;5(8):180171. doi: 10.1098/rsos.180171 (PMC6124126; doi:10.1098/rsos.180171)

Unless otherwise specified at the top of each panel, model parameters were identical as those given in Fig 5: (i.e. exponent $n = 2$, $f_L = 6$ Hz, $f_C = 20$ Hz, and $f_S = 5$ Hz).

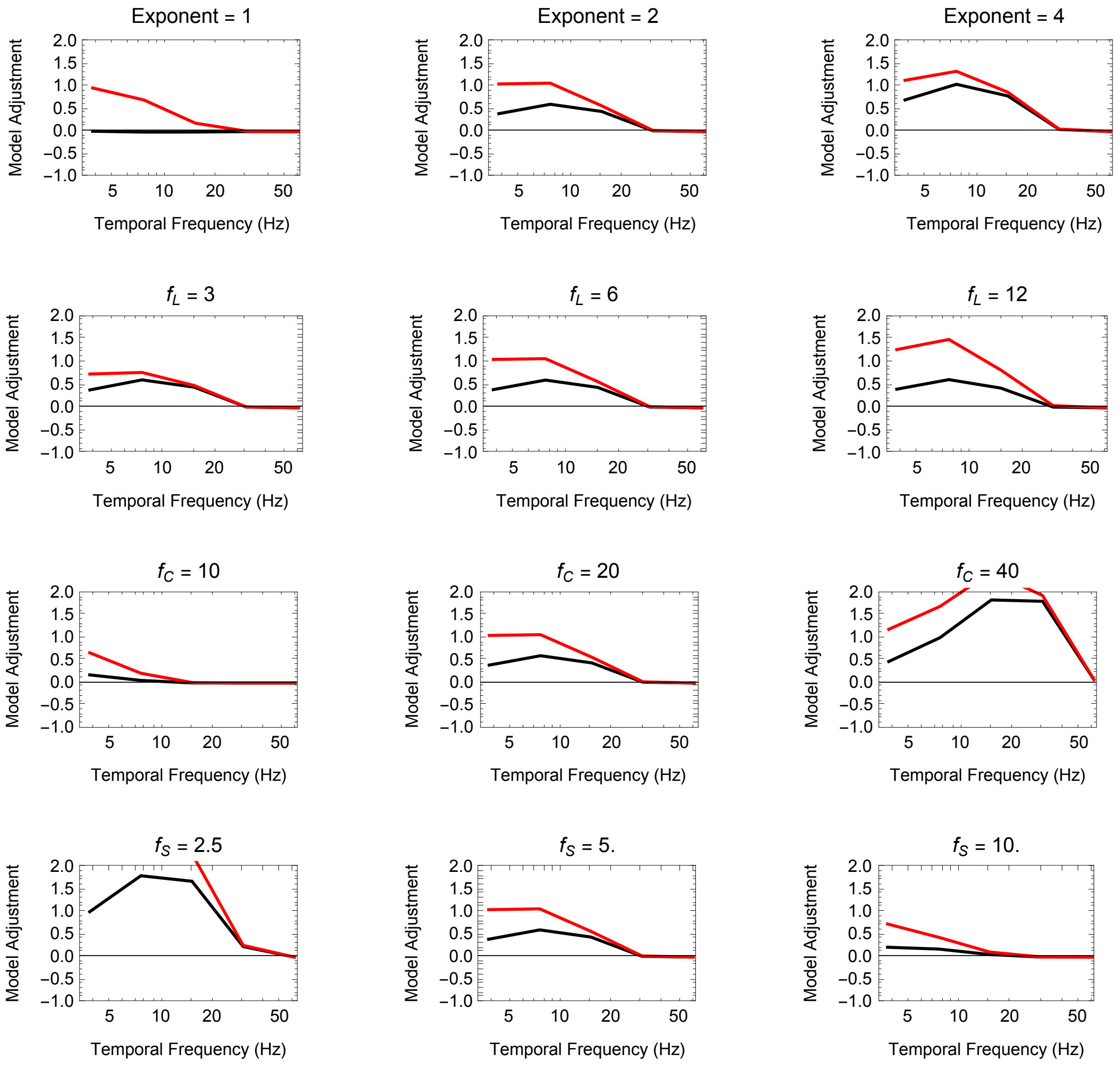

Supplement: Model Behaviour [file rsos180171supp2.pdf]
